# Differential effects of internal tagging depending on depth treatment in Atlantic salmon: a cautionary tale for aquatic animal tag use

**DOI:** 10.1093/cz/zoy093

**Published:** 2018-12-13

**Authors:** Daniel W Wright, Lars H Stien, Tim Dempster, Frode Oppedal

**Affiliations:** 1 Institute of Marine Research, Matre Research Station, Matredal, Norway; 2 Sustainable Aquaculture Laboratory – Temperate and Tropical, School of BioSciences, University of Melbourne, Parkville, Australia

**Keywords:** Atlantic salmon, physostome, Salmo salar, swim bladder, swimming depth, tagging effects

## Abstract

Electronic tags are widespread tools for studying aquatic animal behavior; however, tags risk behavioral manipulation and negative welfare outcomes. During an experiment to test behavioral differences of Atlantic salmon *Salmo salar* in different aquaculture cage types, including ones expected to elicit deeper swimming behavior, we found negative tagging effects depending on whether cages were depth-modified. In the experiment, data storage tags implanted in Atlantic salmon tracked their depth behavior and survival in unmodified sea-cages and depth-modified sea-cages that forced fish below or into a narrow seawater- or freshwater-filled snorkel tube from a 4 m net roof to the surface. All tagged individuals survived in unmodified cages; however, survival was reduced to 62% in depth-modified cages. Survivors in depth-modified cages spent considerably less time above 4 m than those in unmodified cages, and dying individuals in depth-modified cages tended to position in progressively shallower water. The maximum depth that fish in our study could attain neutral buoyancy was estimated at 22 m in seawater. We calculated that the added tag weight in water reduced this to 8 m, and subtracting the tag volume from the peritoneal cavity where the swim bladder reinflates reduced this further to 4 m. We conclude that the internal tag weight and volume affected buoyancy regulation as well as the survival and behavior of tagged fish. Future tagging studies on aquatic animals should carefully consider the buoyancy-related consequences of internal tags with excess weight in water, and the inclusion of data from dying tagged animals when estimating normal depth behaviors.

Electronic tagging studies are rapidly advancing our understanding of how aquatic animals behave (Hussey et al. 2015). For fishes, internally-implanted or externally-attached electronic tags have unraveled detailed knowledge on their behavior to inform the management of wild (Costa et al. 2012; Cooke et al. 2013) and farmed fish populations (Oppedal et al. 2011; Rillahan et al. 2011; Bui et al. 2016). However, it is widely acknowledged that the tagging process and the tags themselves can lead to deviations from normal fish behavior. Potential negative effects include activity level and swimming performance changes, reduced feeding and growth, and compromised survival (Cooke et al. 2011; Thorstad et al. 2013; Jepsen et al. 2015). Tags that alter fish weight in water can also affect fish buoyancy (Perry et al. 2001), which may have cascading ramifications on the behavior of tagged individuals. This is especially concerning when internal and external electronic tags are used to assess depth-related fish behavior and inform management decisions for wild and cultured fishes.

Negative fish buoyancy can result from excess weight of a tag (Gallepp and Magnuson 1972; Perry et al. 2001; Pflugrath et al. 2012). In shallow laboratory tanks (<0.5 m deep), physoclistous, and physostomous fish compensate for added tag weight by increasing their swim bladder gas volume (Gallepp and Magnuson 1972; Fried et al. 1976; Perry et al. 2001; Pflugrath et al. 2012). Physoclists use the *rete mirabile* to internally secrete gases to fill their swim bladder, whereas physostomes gulp air from the surface to fill their swim bladder through a pneumatic duct connected to their esophagus. For physostomous juvenile Chinook salmon in shallow tanks, Perry et al. (2001) noted a proportional increase in swim bladder volume of ∼40–80% (e.g., a relative swim bladder volume increase from 6 to 8.4–10.8%) with tag: fish weight ratios of 3.2–9.4%. Following Boyle’s law, the swim bladder volume of a fish must increase with depth to maintain neutral buoyancy. Therefore, sufficient swim bladder reinflation may become challenging for physostomous fish with added tag weight in water when inhabiting deep water environments. Perry et al. (2001) warned that internal weighted tags may lead to depth behavior modifications of physostomes in environments with large depth ranges. Others have also suggested that implanting tags in the peritoneal cavity could limit swim bladder filling capacity to further restrict buoyancy control and modulate fish depth behavior (Jepsen et al. 2004). However, the effects of implanted tags with excess weight on fish depth behavior have not been thoroughly explored in deep water field studies, despite widespread use of tags to infer depth fish behaviors in wild and aquaculture settings.

In aquaculture research, data logging (e.g., data storage tags [DSTs]) or transmitting tags (e.g., PIT tags and acoustic telemetry tags) are typically used to assess the individual behaviors of fish, with most applications focusing on evaluating swimming depths given the spatial restrictions of fish farms (e.g., Bui et al. 2016; Føre et al. 2017; Johansson et al. 2009; Korsøen et al. 2012a; Rillahan et al. 2011; Stehfest et al. 2017). Previous papers employing these techniques in aquaculture research have seldom considered or assessed whether side effects of tagging are present and could confound data from tagged individuals, despite their mortality being high on occasion (e.g., Stehfest et al. 2017). Aside from pure research applications, a shift toward a precision farming in the expanding marine fish culture sector has seen fish-borne tags in farms become an increasingly attractive option to monitor stock in high resolution and inform farm management decisions (Føre et al. 2017), and these pursuits must consider tagging side effects.

Salmon lice *Lepeophtheirus salmonis* and the amoebic gill disease agent *Paramoeba perurans* are key ectoparasite problems in Atlantic salmon sea-cage aquaculture that are leading to innovations in cage technology. To prevent contact between a mostly surface-dwelling salmon lice larvae and sea-caged Atlantic salmon, many depth-modified cages have been developed, including snorkel (cage with a deep net roof and tarpaulin tube to the surface) (Stien et al. 2016; Oppedal et al. 2017; Wright et al. 2017), skirt (cage with tarpaulin wrapped around upper depths) (Stien et al. 2012, 2018), repeatedly submerged (Dempster et al. 2008, 2009; Korsøen et al. 2009, 2012a), and air dome submerged cages (Korsøen et al. 2012b). These cage modifications consider the provision of surface access or an air space for Atlantic salmon to refill their swim bladders, because long-term submergence causes fast upward tilted swimming from swim bladder deflation along with poor growth and vertebral deformities in Atlantic salmon (Korsøen et al. 2009). Recently, snorkel cages with a freshwater surface layer have also been used to reduce co-occurring amoebic gill disease (AGD) caused by *Paramoeba perurans* (Wright et al. 2017, 2018). Observing depth behavior is crucial in understanding the environmental conditions fish experience and how production can be optimized within these depth-modified cage designs. The extent of swim bladder re-inflation via surface jumps, which are essential for optimal production of Atlantic salmon, is also of special interest in snorkel cages because they tend to reduce jumping frequency (Stien et al. 2016; Oppedal et al. 2017; Wright et al. 2018).

While we set out to test for differences in swimming depth and jumping behavior of individual Atlantic salmon in unmodified cages or depth-modified cages (with seawater-filled snorkel and freshwater-filled snorkels) using DSTs, we observed significant tagging-related mortality depending on the depth treatment applied. Our initial prediction was that the depth-modified cages which restrict surface access would push fish to swim deeper and jump less frequently than in unmodified cages (Stien et al. 2016; Oppedal et al. 2017; Wright et al. 2018). With a general pattern of deep swimming in daylight periods and shallow swimming in dark night periods (reviewed by Oppedal et al. 2011), we were interested in differences in day and night swimming and jumping behavior. However, tagging effects became the focus of this study which we tracked by assessing survival of tagged individuals and the depth behavior changes between survivors and dying individuals.

## Materials and Methods

### Study design

**Figure 1.**
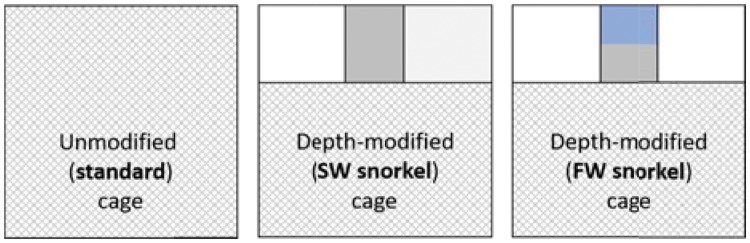
Schematic of an unmodified standard cage and depth-modified FW and SW snorkel cages. All cages were 12 × 12 m square and 12 m deep, although a 4 m deep net roof opening to a 3 × 3 m square and 4 m deep tarpaulin tube to the surface was fitted into snorkel cages (tarpaulin tube represented by grey shading). This restricted fish from accessing netted cage sections (cross-hatched) in the upper part of depth-modified cages. FW snorkels had a constant supply of freshwater added so a surface freshwater layer was created (solid blue shading).

At the Austevoll Institute of Marine Research sea-cage farm facility (Western Norway: 60°N 5.3°E), nine cages (12 × 12 m square, 12 m deep) each held 2000 fish in late October 2016 for 3 months (autumn to winter). These consisted of triplicate depth-modified freshwater-filled (FW) snorkel, seawater-filled (SW) snorkel and unmodified standard cages (Figure 1). The dimensions of the 100% watertight tarpaulin snorkel tubes were 3 × 3 m square, 4 m deep. Fish were fed to excess by automated screw pellet dispensers. SW snorkels were constantly filled with seawater from 4 m depth via a pumping system (135 L min^−1^ pump, Xylem Water Solutions) and FW snorkels were continuously filled with ozone-treated municipal freshwater which created a stable freshwater layer (see Wright et al. 2018). Tagging by a single surgeon was conducted on six fish in each of the nine cages (54 fish tagged in total) from 17 to 19 January 2017. We caught fish by stopping feeding a day prior, dropping a hoop net, feeding, pulling up the hoop net, and transferring groups of fish to be tagged via a hand net into a seawater tank with constant flow. Fish weight (mean ± *SD*: 306 ± 35 g; range: 245–395 g) and length in TL (mean ± *SD*: 30.6 ± 0.2 cm; range: 27.5–32.3) were measured. Tags used (DST-milli-F, 39.4 × 13 mm, 13 g in air, and 5.0 g in water, 0.03% depth resolution and 0.06% depth accuracy, Star-Oddi) represented a mean ± *SD* of 4.3 ± 0.5% of fish weight in air, with a range from 3.2 to 5.3% of fish weight in air. One replicate FW snorkel cage was discounted from all analyses due to inconsistencies in its management (Wright et al. 2018). All stock harbored heavy salmon lice infestations, with mean ± s.e. total lice numbers of 30.3 ± 2.2, 21.8 ± 2.9, and 27.9 ± 2.9 in control, SW snorkel and FW snorkel cages based on lice counting using 20 untagged fish per cage on 31 January at study completion.

### Tagging

We transferred fish into an anesthetic seawater bath (8°C) of metomidate (0.01 g L^−1^) for a mean ± *SD* of 2: 47 ± 1:14 min, ranging from 1–5 min until sedated. Fish were then moved and inverted into a cradle and anesthesia continued by gravity-feeding anesthetic seawater via a tube onto the gills, with flow adjusted by a valve. In this position, fish underwent surgery involving: 1) an incision of 2–3 cm (in the center of the ventral plane of the fish slightly further back from the pectoral fins and continuing posteriorly), 2) implantation of the DST (dome end first, angled posteriorly once half-inserted and when fully-inserted pushed in the same direction past the incision point), and 3) two sutures to close the incision wound (half-moon needle and resorbing thread). A T-bar tag was also inserted through the epidermis layer adjacent to the dorsal fin for identification at retrieval. Equipment was sterilized using 100% alcohol between fish. Surgery lasted a mean ± *SD* of 4: 42 ± 0: 57 min and ranged from 3 to 8 min. Afterwards, fish were returned to a seawater tank and monitored for recovery, determined by upright orientation and normal swimming behavior. Recovery took a mean ± *SD* of 10:18 ± 4:39 min and ranged from 4 to 27 min. Following an additional recovery period of 1 h, fish were returned to cages from tanks by hand netting.

### Tag data preparation

Tags recorded depth every second and temperature every 4:15 min. Logging commenced immediately prior to tagging fish and ceased 14–16 days later (1–3 February) when tagged individuals were recaptured. Retrieval of tagged fish involved removing snorkels in the case of FW and SW snorkel cage types, raising the cage net to ∼2 m depth and hand netting the fish with conspicuous T-bar tags from the caged population. Snorkels were removed from 31 January, so data from all tagged individuals were excluded after 30 January. Mortalities were checked three times per week during dead fish collections by farm staff, at which point any fish with T-bars were removed and their DST tags retrieved. Time of death for tagged individuals was determined from the point that fish fell to the cage bottom with no further depth changes.

For overall depth datasets, data points at 4:15 min intervals were selected. Data from a post-surgery recovery period of 24 h after fish were added to cages was censored from analysis, and consequently datasets were reduced to 11–13 days (18, 19, or 20 January until 30 January). Mean sunrise and sunset times of 09:05 and 16:34 were used to define day and night periods. Tags that 1) were not retrieved (1 in SW snorkel and 1 in standard cage), 2) were from fish that died within the 24 h recovery period (1 in FW snorkel and 1 in SW snorkel), and 3) malfunctioned (1 in SW snorkel cage), led to 11 of 12 fish from FW snorkel cages, 15 of 18 fish from SW snorkel cages and 17 of 18 fish from standard cages being analyzed in depth datasets.

For jump datasets, a correction (mean of −0.22 m, range of 0.00 to −1.18 m) was applied to depths based on readings of individual tags submerged at the water’s surface in testing prior to the trial, which improved the accuracy of jump detections. Only individuals alive at study completion were included in jump analyses. In addition, datasets were not built for fish entering the top 0.25 m (based on corrected data) of the water column for extended periods, because their jumps were impossible to detect from depth changes at these times. In total, 5 of 12 fish from FW snorkel cages, 8 of 18 fish from SW snorkel cages and 7 of 18 fish from standard cages were analyzed for jump datasets.

Jump sequences were identified and captured using the following rules: 1) fish must have entered the top 2 m (no fish were recorded changing their depth faster than 4 m sec^−1^ to allow a jump without being detected in the top 2 m); 2) the depth changes prior to or after a suspected jump needed to be fast enough to allow them to reach the surface (e.g., a fish moving at 1.2 m sec^−1^ from 1.6 to 0.4 in the previous second could have jumped between 0.4 and 0.6 m within the following second); and 3) jump sequences started from when the first positive depth change was recorded before the jump until the last negative depth change after the jump. Jumps were recalculated to jumps day^−1^ separated into day and night periods for individual fish (e.g., 28 jumps at night ÷ 10 nights = 2.8 jumps day^−1^ during night periods). This accounted for differences in the number of days fish were monitored. For each jump, a maximum vertical speed was also determined from the maximum depth change during a second interval.

**Figure 2.**
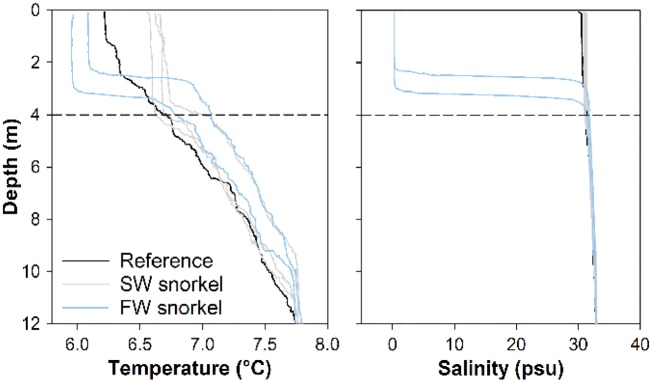
Temperature (left) and salinity profiles (right) in SW (grey) and FW snorkel cages (blue), and at a reference location indicative of conditions in standard cages (black line) on 24 January 2017. The snorkel depth (dotted horizontal line) at 4 m is displayed.

To help explain tagged fish behavior, temperature and salinity depth profiles were recorded daily via a centrally located welfare meter programmed to profile a Conductivity Temperature Depth (CTD) device between 0 and 15 m at 12 pm daily (APB5, SAIV, Bergen, Norway). In the 2-week study period, temperature and salinity levels ranged from 4.7°C to 8.3°C and 28.7 to 33.8 psu. Temperature and salinity depth profiles were also conducted at the reference location and within each FW and SW snorkel cage between 0 and 12 m with a CTD device (SD204, SAIV) on 24 January 2017. Deviance from reference conditions occurred in the 4 m deep snorkels; with warmer more saline water filled from 4 m depth inside SW snorkel cages and a cooler freshwater layer permanently created in the top 2–3 m within FW snorkel cages (Figure 2).

### Statistical analysis

For multiple comparisons of tagged fish survival between cage types (treatments), we used Kaplan–Meier log-rank analysis and Holm–Sidak post-hoc tests in Sigmaplot 13.0 (Systat Software Inc. 2014). To assess whether fish weight differed between surviving and dying tagged individuals in snorkel cages, Linear Mixed-effect Models (LMMs) were performed. Here, fish weight was used as the response variable, survival status as the predictor variable and cage was added as a random effect. Proportions of surviving untagged individuals in each cage over the study period were compared between treatments using a generalized linear model (GLM) analysis, with surviving individuals as the response variable and treatment as the predictor variable and a quasibinomial distribution. Mixed-effect model analysis was not used here because cage was the level of replication.

Mean daytime and night time swimming depths were compared between treatments using LMMs, with swimming depth as the response variable, treatment as the predictor variable and cage as a random effect. We also tested for differences in daytime and night time swimming depths between surviving and dying tagged individuals in snorkel cages using LMM analyses with swimming depth as the response variable, survival status as the predictor variable, and cage as a random effect. Datasets for fish that died were filtered to contain data from −4 to 0 days before death, which maximized the number of individuals contributing to data for each time point. We used Spearman’s correlation tests to determine whether relationships existed between both day and night average swimming depths relative to days before death for dying individuals in snorkel cages. These analyses were repeated for day and night average swimming depths and monitoring days for surviving individuals in snorkel cages and control cages. Monitoring days between 20 and 30 January were used, again to maximize individuals contributing to each time point.

To compare daytime and night time jumping frequency (jumps day^−1^) of surviving tagged individuals, GLM analysis was carried out with treatment as a factor and a quasipoisson distribution. We also assessed differences in night time jumping speed (proportions of jumps with a maximum vertical jumping speed of >4 BL sec^−1^) between cage types via GLM analysis with treatment set as a factor and a quasibinomial distribution. Day time jumping speed differences between cage types were not assessed because variances were often at or near zero due to fast jumping by most tagged fish (Table 1). Mixed-effect model analysis of jumping behavior was not possible due to low fish numbers in some cages. For LMM and GLM analyses, full models were compared with corresponding null models excluding the predictor variable in analysis of deviance tests producing χ^2^ and *P*-values. Significant LMM results were followed by least-squares means post-hoc tests for multiple comparisons, whereas significant GLM model results were followed by Tukey HSD post-hoc tests. Excluding Kaplan-Meier log-rank analysis, tests were performed in R 3.1.0 (R Core Team 2016). Error distributions of all models were assessed visually for non-normality and heterogeneous variance.

**Table 1.** Summary statistics of swimming depth and jumping behavior of tagged individuals that survived and died between standard, FW snorkel and SW snorkel cages. Jump datasets were not created from fish that died in FW and SW snorkel cages

Treatment	Time < 4 m depth (%)	Day depth (mean ± s.e.)	Night depth (mean ± *SE*)	Day jumps day^−^^1^ (mean ±*SE*)	Night jumps day^−^^1^ (mean ±*SE*)	Day jumps > 4 BL sec^−^^1^ (%)	Night jumps > 4 BL sec^−^^1^ (%)
Standard (survived)	74	−4.0 ± 0.2	−3.1 ± 0.2	1.3 ± 0.8	2.1 ± 0.7	24	22
SW snorkel (survived)	29	−7.4 ± 0.1	−4.8 ± 0.4	0.6 ± 0.1	2.1 ± 0.5	96	23
FW snorkel (survived)	4	−8.5 ± 0.6	−7.1 ± 0.5	0.5 ± 0.1	0.3 ± 0.1	100	94
SW snorkel (died)	53	−6.5 ± 0.2	−3.1 ± 0.6				
FW snorkel (died)	9	−6.9 ± 0.6	−5.6 ± 0.2				

## Results

### Survival

Tagged fish survival was affected by treatments (Kaplan–Meier survival test: log-rank statistic = 9.6, *df =* 2, *P = *0.008). Compared with standard cages (17 of 17 fish or 100%), survival was lower in FW snorkel (8 of 12 fish or 66.6%, Holm–Sidak post-hoc test: *P = *0.02) and SW snorkel cages (10 of 17 fish or 58.8%, Holm–Sidak post-hoc test: *P = *0.004). No difference in tagged fish survival was found between FW and SW snorkel cages (Holm–Sidak post-hoc test: *P = *0.4). For the tagged fish in snorkel cages confirmed to have died or survived, weight appeared to be affected by survival status from LMM analysis (mean ± s.e. weight of 301 ± 10 for dying vs 306 ± 10 for surviving individuals, LMM Analysis of Deviance test: *χ^2^* = 8.4, *df* = 1, *P* = 0.003). Over the study period from 18 to 30 January, survival of caged populations (not including tagged individuals) was 99.6, 99.3, and 99.7%, respectively, in standard, SW snorkel, and FW snorkel cages, and did not differ between treatments (GLM Analysis of Deviance test: *χ^2^* = 0.06, *df =* 2, *P = *0.3).

### Swimming depth

**Figure 3.**
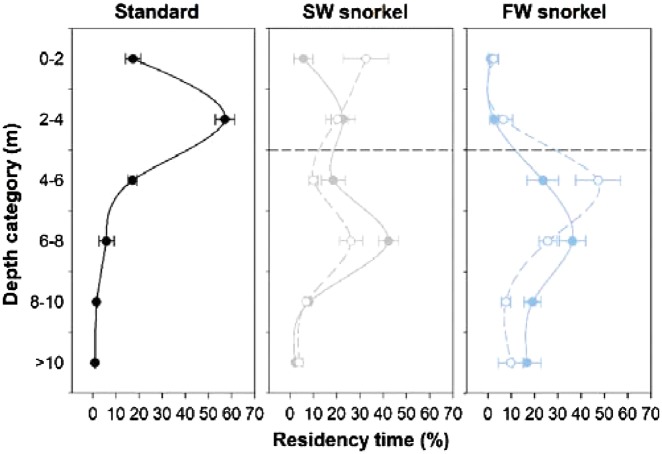
Mean ± *SE* proportions of times tagged salmon swam in 2 m depth intervals among individuals in standard (left, black), SW snorkel (middle, grey), and FW snorkel fish (right, blue lines). Solid and dotted lines represent depths of surviving and dying individuals, respectively. Data were pooled from 1 day post-release between 18–20 January and 30 January 2017.

**Figure 4.**
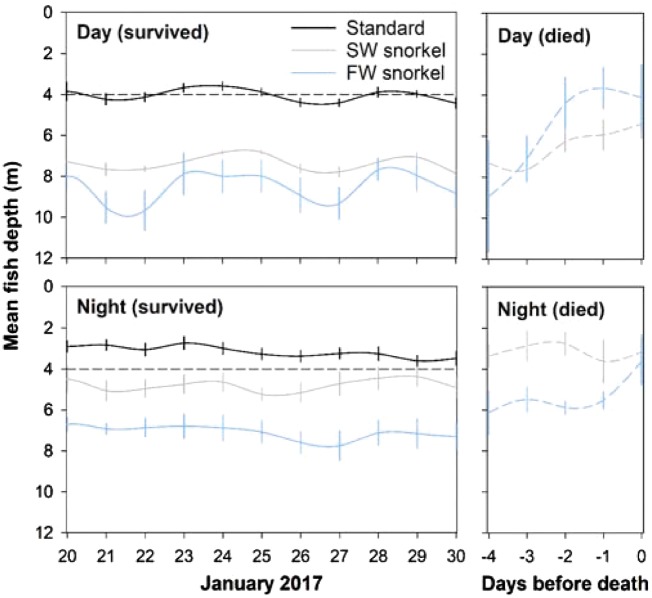
On the left, mean depth of tagged surviving individuals on each study day separated between day and night periods for standard (black), SW snorkel (grey), and FW snorkel fish (blue solid line) from 20 to 30 January 2017. On the right, mean depth of dying individuals on −4 − 0 days before death separated between day and night periods for SW snorkel (grey) and FW snorkel fish (blue dashed line).

Swimming depth of tagged individuals surviving the experiment duration (termed survivors hereafter) was affected by treatments during day (LMM Analysis of Deviance test, *χ^2^* = 20.9, *df =* 2, *P *<* *0.0001) and night periods (LMM Analysis of Deviance test, *χ^2^* = 21.2, *df =* 2, *P *<* *0.0001). Survivors swam shallower in standard cages than in SW and FW snorkel cages and FW snorkel cages in both day and night periods (Least-squares means post-hoc tests, *t *≥* *3.6 *P *≤* *0.003, Table 1, Figures 3 and 4). In addition, survivors swam shallower in SW than FW snorkel cages in night periods (least-squares means post-hoc test, *t = *4.2, *P = *0.0005, Table 1, Figure 3 and 4). Survivors in FW snorkel cages spent 1% of the time or 16:52 ± 0:43 min day^−1^ in the top 2 m of snorkels, where a permanent freshwater layer was present. Within snorkel cages, survival status also affected swimming depth in day and night periods (LMM Analysis of Deviance tests, *χ^2^* ≥ 4.9, *df =* 1, *P *≤* *0.03), with individuals dying during the experiment (dying individuals hereafter) entering snorkels for longer periods than survivors (Table 1, Figures 3 and 4).

Day time average depths of dying individuals in snorkel cages were shallower as time before death decreased (Spearman’s correlation test, r_s_ = 0.60, *df =* 29, *P = *0.0005, Figure 4). In contrast, night time average depths of dying individuals in snorkel cages remained unchanged with time before death (Spearman’s correlation test, *r_s_* = 0.17, *df =* 30, *P = *0.3, Figure 4). Time also had no influence on the average depth of survivors in snorkel cages within day (Spearman’s correlation test, r_s_ = −0.02, *df =* 185, *P = *0.8) or night periods (Spearman’s correlation test, *r_s_* = −0.01, *df =* 185, *P = *0.9) or survivors in control cages in the day (Spearman’s correlation test, *r_s_* = −0.12, *df =* 185, *P = *0.09, Figure 4). However, in night periods, survivors in control cages tended to descend into slightly deeper water later in the trial (Spearman’s correlation test, *r_s_* = −0.23, *df =* 185, *P = *0.002, Figure 4).

### Surface jumps

**Figure 5.**
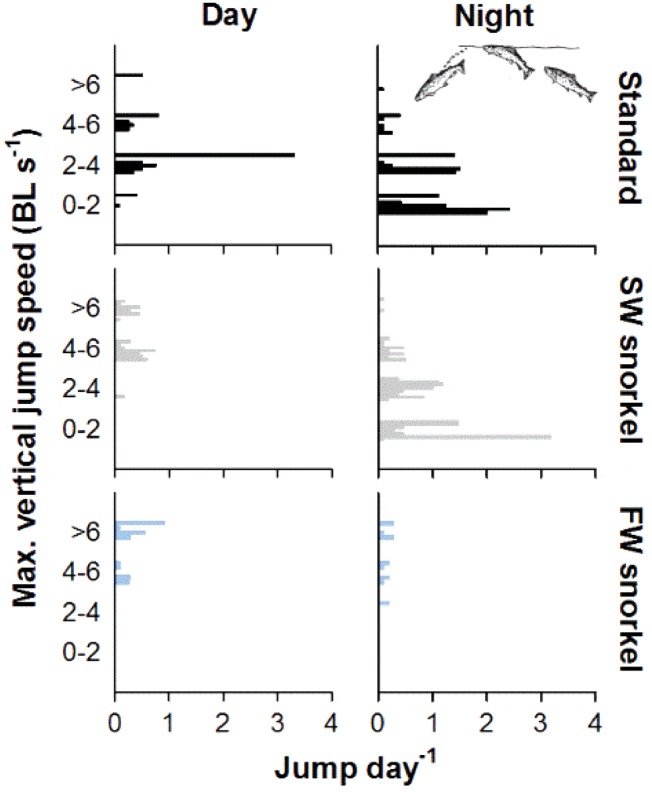
Individual jumps day^−1^ plotted against maximum jumping speed in the vertical plane at 2 BL sec^−1^ intervals for surviving tagged salmon in standard (black), SW (grey), and FW snorkel cages (blue bars) separated between day and night periods. *Source:* Image modified from Korsøen et al. 2012b.

Jumping frequency of survivors was similar between treatments in day periods (GLM Analysis of Deviance test, χ^2^ = 3.0, *df =* 2, *P = *0.2), but differed at night (GLM Analysis of Deviance test, χ^2^ = 9.8, *df =* 2, *P = *0.002, Table 1, Figure 5). At night, FW snorkel fish jumped at least 7 times less often than standard (Tukey HSD post-hoc test, z = 2.4, *P = *0.03) and SW snorkel fish (Tukey HSD post-hoc test, z = 2.4, *P = *0.04), whereas no differences were seen between standard and SW snorkel fish (Tukey HSD post-hoc test, z = 0.4, *P = *0.9, Table 1, Figure 5). During the day, maximal vertical jumping speeds by survivors were faster in SW and FW snorkel cages than standard cages (Table 1 and Figure 5). At night, jumping speed differed between treatments (GLM Analysis of Deviance test, χ^2^ = 8.3, *df =* 2, *P = *0.0001), with FW snorkel fish jumping faster than standard (Tukey HSD post-hoc test, z = 2.8, *P = *0.02) and SW snorkel fish (Tukey HSD post-hoc test, z = 2.7, *P = *0.02), but no difference between standard and SW snorkel fish (Tukey post-hoc test, z = 0.8, *P = *1.0, Table 1 and Figure 5).

## Discussion

### Tagged salmon mortality in depth-modified cages

Atlantic salmon in depth-modified cages swam deeper and were more affected by tagging effects than in unmodified cages. Significant decreases in survival of tagged fish compared with overall caged populations were recorded in depth-modified cages, but not in unmodified cages. Tagged fish in all cage types performed jumps related to swim bladder filling (Dempster et al. 2011), which allows fish to compensate for the tag weight (Gallepp and Magnuson 1972; Fried et al. 1976; Perry et al. 2001). However, we suggest that tagged fish in deep water (e.g., below 4 m depth) could not compensate sufficiently to attain neutral buoyancy and that maximum swim bladder volumes likely used inside a peritoneal cavity holding a large tag contributed to the mortalities in depth-modified cages.

The lower survival of tagged fish in depth-modified than standard cages suggested tagging effects were related to buoyancy regulation. We estimated maximum neutral buoyancy depth of fish in our study, based on previous experiments on Juvenile Chinook Salmon (Pflugrath et al. 2012). Estimated maximum neutral buoyancy depth changed from 22.0 m in seawater for untagged fish to 8.4 m for tagged fish if only factoring in the tag weight or 4.2 m if factoring in tag weight and swim bladder volume lost due to the tag volume (see Supplementary Material 1). If these values are accurate, tagged individuals were more likely to experience negative buoyancy and maximum swim bladder volumes in depth-modified cages partially excluding fish from the top 4 m. Under situations where the swim bladder was inflated past its maximum volume minus the added tag volume, internal organs in the peritoneal cavity or the incision made for tag insertion were potentially affected. The combination of negative buoyancy and maximum swim bladder inflation in a peritoneal cavity of restricted volume may have contributed to tagged fish deaths in the depth-modified cages.

Dying tagged individuals in depth-modified cages spent longer periods in surface layers, and used progressively shallower depths in daylight as death approached. Possible explanations include that fish may have selected surface layers to avoid potential negative buoyancy and consequences of maximum swim bladder volumes associated with internal tags with extra weight in water (Perry et al. 2001; Pflugrath et al. 2012). Neutral buoyancy, being more likely in shallow water, would have reduced energy expenditure on fish swimming. Negatively buoyant Atlantic salmon with deflated swim bladders held in submerged cages swim faster than fish in unmodified cages at similar swimming depths, with energy use consequences (Dempster et al. 2009). Shallow water may also have been preferred by tagged fish suffering from infection or other health issues before death (e.g., Toranzo et al. 2005), or becoming subordinate and vertically separated from daytime schools within cages due to deterioration in their condition (Bridger and Booth 2003).

### Tagged salmon behavior in depth-modified cages

While mortalities associated with tagging indicated the potential for tags to affect fish behavior, data from surviving rather than dying individuals were more likely to be representative of untagged individuals. Surviving tagged individuals in depth-modified cages with 4 m deep snorkels swam deeper than those in unmodified cages, although increased residency in the upper layers of SW snorkel cages was detected during night periods. Using echo sounders, Stien et al. (2016) found harvest-sized salmon mostly avoided the 4 m deep SW snorkels during both day and night. Increased residency in 4 m deep SW snorkels in our study could be the result of greater numbers of smaller fish being capable of packing into the snorkel space. Tags with added weight may have also caused shallower swimming behavior than untagged fish that would be monitored by echo sounders. Understanding fish depth behavior in depth-modified cage designs using a combination of echo sounder and tagging methods to track fish depth would be enlightening.

To resolve day and night time jumping behavior of Atlantic salmon, we employed high temporal resolution depth DST tags. Previously, day time only visual observations have been typical (Furevik et al. 1993; Dempster et al. 2011; Bui et al. 2013; Stien et al. 2016; Oppedal et al. 2017) or continuous infrared or PIT telemetry methods have been trialed but can suffer from poor detection efficiencies (Furevik et al. 1993; Korsøen et al. 2012b). We show DST tags to be an alternative method for tracking jump frequency and the maximum vertical speed used during jumps throughout diel cycles. However, the method was not effective when individuals spent extended periods in surface waters as detecting depth changes during jumps from background swimming depths was impossible. Using tags that combine depth recordings with speed, tail fin beat activity, body angle measurements are likely to improve jump detections from DSTs (Tanaka et al. 2001; Watanabe et al. 2008). Our study, suggesting a tendency for slower, more numerous jumps at night, highlights the need to assess diel variation in Atlantic salmon jumping behavior.

Jumping frequency was similar in standard and SW snorkel cages, suggesting that the number of Atlantic salmon swim bladder refilling episodes were comparable regardless of depth modification to cages. Although, it should be noted that jumps may also be performed in response other drivers, such as salmon lice infestations (Samsing et al. 2015; Atkinson et al. 2018). Jumping frequency differed from previous studies showing fewer day time jumps in SW snorkel compared with standard cages (Stien et al. 2016; Oppedal et al. 2017; Wright et al. 2018). The observed higher than expected jumping frequency in SW snorkel cages may be attributable to the negative effects on buoyancy regulation experienced by tagged individuals within this cage type, the different method of jump detection, or the influence of other jumping behavior drivers.

Fish appeared to jump faster through the vertical plane when positioned deeper in the water column, which was typical for FW snorkel fish in both day and night periods and SW snorkel fish during the day. Maximal vertical jumping speeds were conceivably faster in snorkel compared with unmodified cages because they were forced to swim at a steeper angle through the snorkel to the surface. Swimming speeds of up to 2.5 m s^−1^ or 8.6 body lengths s^−1^ were recorded; equivalent to burst swimming speeds measured for ∼50 cm long Atlantic salmon of 8.4 body lengths s^−1^ (Colavecchia et al. 1998). While high vertical jump speeds with maximal swim bladder volumes by tagged individuals may have contributed to negative tagging effects in depth-modified cages, it should be noted that tagged fish in all cage types used high maximal vertical jump speeds at least in some instances.

Freshwater surface layer avoidance was evident from minimal fish residency in surface waters (in the top 2 m for 16 min day^−1^) along with rapid (95% of jumps using > 4 BL sec^−1^) and few jumps (0.8 jumps day^−1^) through a freshwater layer in snorkel cages. Freshwater avoidance by seawater-acclimated post-smolt Atlantic salmon may be explained by fish minimizing osmoregulatory energy expenditure, which is particularly metabolically costly for small fish experiencing salinity variation (Stefansson et al. 2012). Previous studies examining the environmental drivers of Atlantic salmon behavior in sea-cages indicate salinity is a weak driver of swimming depth compared with light and temperature, with the possible exception of recent post-smolts (reviewed by Oppedal et al. 2011). To date, studies have only been conducted in sea-cage environments where surface salinities have reached 15 (Johansson et al. 2006, 2007), 13 (Johansson et al. 2009), 8 (Oppedal et al. 2007), and 4 psu (Oppedal et al. 2001). Therefore, salinity may only become an important variable when a pure freshwater layer is present in the water column. However, it is also possible that the slightly cooler temperature of the freshwater layer contributed to avoidance by Atlantic salmon, with thermal preferences known to heavily influence salmon swimming depth behavior (Oppedal et al. 2011). Future studies are needed to better understand trade-offs between major environmental drivers of sea-caged salmon behavior when strong salinity gradients are present.

### Recommendations for future tagging studies

Our experiment provides an example of internal electronic tagging negatively affecting an aquatic animal depending on the depth treatments applied. The use of depth-modified cages, which forced deeper swimming by tagged physostomous Atlantic salmon and likely caused negative buoyancy and maximal swim bladder volumes within a peritoneal cavity holding a large tag, was the suspected cause of their reduced survival and shallower swimming before death. We provide a range of recommendations for future physostomous fish tagging studies so negative animal welfare outcomes are avoided and the reliability of depth measurements from aquatic animals is improved.

Lower tag: fish weight ratios or tags with no added weight in water could be used to minimize extra fish weight in water (Perry et al. 2001). While others have suggested tag: fish weight in air ratios of up to 12.7% are satisfactory for Atlantic salmon (Newton et al. 2016), our results suggest that the general 2% rule should be a minimum standard for physostomous fishes (Jepsen et al. 2004). Furthermore, rules specifying tag: fish weight in water ratios would be more appropriate for limiting tagging effects on fish buoyancy (Perry et al. 2001).

Lower tag volumes inside the peritoneal cavity could be employed to diminish tagging effects on maximum swim bladder volume, internal organs and the incision wound. Using external tags would also avoid adding internal tag volume or introducing a tag incision wound, however they may present other tagging issues. As an example, increased mortality or tag loss occurs when using external compared with internal tags to study wild Chinook salmon (Brown et al. 2013). For internal tagging, a post-surgery recovery period in shallow cages or tanks, in which tagged fish would not need maximum swim bladder volumes to achieve neutral buoyancy whereas the incision heals (e.g., Atlantic salmon held for 3–12 weeks in 5 m deep cages before transfer to deeper cages), may also be beneficial (Bui et al. 2016; Korsøen et al. 2012a).

Altered behavior by dying tagged individuals in our study also indicated that data from dying tagged individuals should be discounted for a defined period of altered behavior before their death or completely to ensure the quality of depth information from fish tagging studies.

Awareness of buoyancy effects from animal tags could be important beyond physostomous fishes. For example, adding excess weight in water similarly raises the maximum neutral buoyancy depth of air-breathing animals (e.g., loggerhead turtles) (Minamikawa et al. 2000), and would increase negative buoyancy in fishes without a swim bladder. However, physoclistous fishes would more easily compensate for additional tag weight in water via internal swim bladder filling at depth. We hope that the described experiment herein serves as an important cautionary tale for others seeking to use electronic tags to understand normal behaviors of aquatic animals.

## Supplementary Material

zoy093_Supplementary_Data
