# Supplementary material for: Differential effects of internal tagging depending on depth treatment in Atlantic salmon: a cautionary tale for aquatic animal tag use
Source: Curr Zool. 2018 Dec 13;65(6):665–73. doi: 10.1093/cz/zoy093 (PMC6911844; doi:10.1093/cz/zoy093)
Supplement: zoy093_Supplementary_Data [file zoy093_supplementary_data.docx]

**Supplementary Figure 1.** Survival of tagged individuals in each of the standard (black), SW snorkel (grey) and FW snorkel cages (blue lines) over the study period. Individuals not known to have died were censored.





**Supplementary Material 1.** Rationale and equations used to estimate maximum depth of neutral buoyancy for tagged Atlantic salmon in the present trial.

Maximum neutral buoyancy depth for untagged juvenile chinook salmon of 44.8 g weight has been calculated to be 6.7 m in freshwater (Pflugrath et al. 2012). This value was arrived at from an experiment adding externally attached weights to the fish until they were no longer able to attain neutral buoyancy (based on visual assessment) in shallow tanks. We estimated the maximum swim bladder volume for juvenile chinook salmon (*V_SBm_*) to be 4.54 cm^3^ from Pflugrath et al. (2012). We then determined how implanting internal tags, at the same weight and volume ratios as in our study, affected the maximum neutral buoyancy depth (*D_mnb_*) of juvenile chinook salmon and potentially Atlantic salmon post-smolts using equations 1–3.

Firstly, the swim bladder volume needed for neutral buoyancy (*V_SBn_*) was calculated by factoring in a tag weight in air (*W_ta_*) and volume (*V_t_*) for juvenile salmon at the same 4.2% tag: fish weight in air ratio (*W_ta_* = 1.9 g and *V_t_* = 1.01 cm^3^) and water density (*ρ_w_*) of 1.00 or 1.025 g cm^-3^ for freshwater and seawater (Perry et al. 2001):

*V_SBn_* = [ (*W_fa_* + *W_ta_* ) / *ρ_w_*) ] – (*V_f_* + *V_t_*) (1)

Secondly, maximum swim bladder pressure (*P_SBm_*) was determined from Boyle’s law (typically P_1_V_1_ = P_2_V_2_), where atmospheric pressure (*P_a_*) was 101.3 kPa:

*P_SBm_* = (*P_a_* × *V_SBm_*) / *V_SBn_* (2)

Thirdly, *D_mnb_* was found using a rate of pressure increase with water depth (*∆P_d_*) of 9.98 or 10.00 kPa m^-1^ in freshwater or seawater (Pflugrath et al. 2012):

*D_mnb_* = (*P_SBm_ - P_a_*) / *∆P_d_* (3)

Based on these calculations, juvenile chinook salmon and potentially Atlantic salmon post-smolts have a *D_mnb_* of 6.7 m in freshwater or 22 m in seawater. Added tag weight at equivalent ratios used in our study reduced the *D_mnb_* to 2.6 m in freshwater and 8.4 m in seawater. If the maximum swim bladder volume was reduced by tag volume in the peritoneal cavity space (*V_SBm_* – *V_t_*), then *D_mnb_* would have been -0.3 m in freshwater (fish always negatively buoyant) or 4.2 m in seawater. These estimated values of *D_mnb_* would be applicable to post-smolt Atlantic salmon in our study if: a) Atlantic and chinook salmon have equivalent fish densities and swim bladder sizes, b) fish density is identical between juveniles and post-smolts, and c) swim bladder size increases proportionally to fish size for juveniles and post-smolts.
